# Oily Fish and Omega-3s Across the Life Stages: A Focus on Intakes and Future Directions

**DOI:** 10.3389/fnut.2019.00165

**Published:** 2019-11-12

**Authors:** Emma Derbyshire

**Affiliations:** Nutritional Insight Limited, London, United Kingdom

**Keywords:** oily fish, omega-3 fatty acids, dietary intakes, supplementation, health

## Abstract

**Background:** There is a tendency to report oily fish intakes for adults collectively. This means that certain population groups tend to be overlooked. The purpose of the present article is to derive and evaluate oily fish and omega-3 intakes across the lifespan.

**Methods:** A secondary analysis of the UK National Diet and Nutrition Survey (years 2008–2016) was undertaken. Data from *n* = 2,949 participants ≥4 years was analyzed. Alongside this, data was extracted from surveys published within the last 5-years reporting omega-3 intakes.

**Results:** Overall, only a quarter (25.2%) of the UK population are oily fish consumers. Amongst those eating oily fish only 7.3% of children, 12.8% of teenagers, and 15.6% of young adults (20–29 years) met oily fish recommendations. Mean intakes of oily fish ranged between 3.4 and 19.1 g/day. Females aged 30–39 and 60–69 years had significantly lower daily oily fish intakes than males (*P* = 0.05 and *P* = 0.049) although their intakes were higher than men in their fifties (*P* = 0.048). Between 2008 and 2016 oily fish intakes have remained relatively stable although a significant decline was seen amongst those aged 50–59 years (*P* = 0.048). Survey data (*n* = 10 publications) showed that EPA and DHA intakes were consistently lower than guidelines, with children, teenagers, females, and pregnant women having some of the largest dietary gaps.

**Conclusions:** Younger generations, women of childbearing age and pregnant mothers appear to be at particular risk of oily fish and omega-3 shortfalls. Declining EPA and DHA profiles of farmed fish and plant-based food movements are only likely to exacerbate already inadequate intakes. Urgent public health campaigns are needed to improve UK intakes, which should include a combined approach of dietary and supplemental sources.

## Introduction

Oily fish is a valuable source of energy, protein, fat and long-chain omega-3 polyunsaturated fatty acids, mainly taking the form of Eicosapentaenoic Acid (EPA) and Docosahexaenoic acid (DHA), which play a valuable role in disease prevention and health promotion (1, 2). Within the diet oily fish is an important provider of EPA and DHA which is attributed to the fact that microalgae rich in EPA and DHA is the main food of many fish (3, 4). However, there is a generic tendency to under consume oily fish which means that meat (particularly poultry) and eggs are some of the main dietary contributors (5, 6).

Omega-3 fatty acids (*n*-3 FAs) have been a focal point of interest to scientists for many years (7). Alpha-linoleic acid (ALA) is an “essential fatty acid” as it cannot be produced by the human body or higher organisms and is converted into other longer *n*-3 FAs including eicosapentaenoic acid (EPA) and docosahexaenoic acid (DHA) (8). *n*-3 FAs are a heterogeneous group of fatty acids which have a double bond located between the third and fourth carbon atoms from the methyl end (7). As the human body does not produce longer *n*-3 FAs efficiently it is necessary to obtain suitable amounts through dietary sources (9).

**Figure 1:**
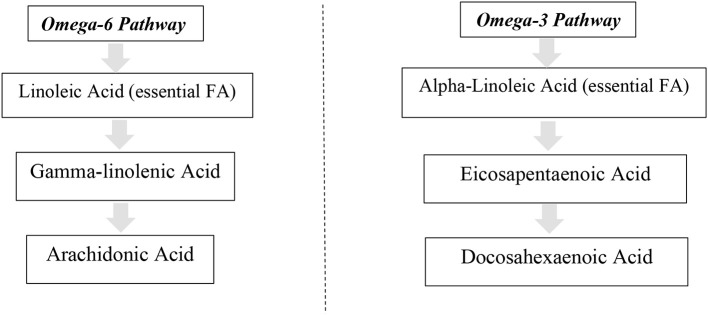
Metabolic pathways of omega-6 and 3 fatty acids.

As shown in Figure 1 the *n*-3 FA alpha-linoleic acid (ALA) is the only real “essential” FA as it is required to yield EPA and DHA which, in turn, produce anti-inflammatory eicosanoids (8). Linoleic acid (LA) is the essential omega-6 fatty acid (*n*-6 FA), which yields arachidonic acid (ARA) and produces eicosanoids that can exacerbate inflammation (8). Unfortunately, many factors can affect the conversion of ALA to DHA. This process tends to be more effective in infants and young women compared with young men with the inefficient conversion further emphasizing the importance of obtaining preformed DHA from dietary and supplementary sources (10). It has been proposed that higher rates of conversion to DHA amongst women may be due to their higher requirements during pregnancy and lactation (11).

Previous information suggests that human beings once evolved on diets with a balanced 1:1 ratio of *n*-6 to *n*-3 FAs (12). Unfortunately, in modern Westernized diets this once balanced ratio appears to have shifted up to 15/1-17/1 (12). Modern diets now provide around 80 to 90% of polyunsaturated fatty acids as *n*-6 LA and are lacking in *n*-3 FAs—creating an unnatural balance of LA to α-linoleic acid, EPA and DHA (13). Rising soybean oil intakes during the Twentieth century are thought to be one driver behind rising *n*-6 LA intakes and reduced EPA and DHA tissue concentrations (14).

Oily fish and their associated *n*-3 content have been linked to an array of health benefits. Increased *n*-3 FA intakes elevate EPA and DHA levels in blood lipids, modifying the structure of cell membranes and membrane proteins which have extended roles in cell signaling and gene expression (15). DHA plays a key role in brain and eye development while EPA and DHA together alter cell and tissue receptiveness in a manner that provides optimal conditions for development, growth and the preservation of health (15). Unfortunately *n*-3 status is generally poor—a large global survey of *n*-3 profiles showed that Europe has very low blood levels (<4%) of EPA+DHA in erythrocytes, increasing chronic disease risk (16).

*n*-3 dietary guidelines between countries are also not fully aligned (Table 1). Mostly, two kinds of dietary reference values are derived for *n*-3 FAs, the Adequate Intake as established by the European Food Safety Authority (17), and the acceptable macronutrient distribution range, set by Food and Agriculture Organization/World Health Organization (18). Other guidance has also been compiled by the World Association of Perinatal Medicine Dietary Guidelines Working Group (19). Regarding fish guidance in the UK advice to eat two weekly (140 g) portions of fish, of which one should be oily has been set in place since 2004 (20).

**Table 1:** n-3 FA recommendations.

**Population group**	**EFSA** **(****17****)**		**FAO/WHO** **(****18****)**		**Koletzko et al. (19)**
	**EPA**	**DHA**	**EPA**	**DHA**	**DHA**
Adults	250 mg/d		300 mg/d (of which 200 mg should be DHA)		–
Pregnancy	–	+100–200 mg/d	300 mg/d (of which 200 mg should be DHA)		200 mg DHA
Lactation	–	+100–200 mg/d	300 mg/d (of which 200 mg should be DHA)		200 mg DHA

Data from the United Kingdom (UK) National Diet and Nutrition Survey Rolling Programme (NDNS-RP) has shown few changes in oily fish intakes over the last 9 years (between 2008/09 and 2016/17) (21). The percentage of children aged 11–18 years consuming oily fish did increase by 10 percentage points between 2008/09 and 2016/17 although mean intakes remained well beneath recommended levels across all age groups (21). Whilst this survey provides valuable data for children aged 11 to 18 years and adults aged 19 to 64 years, intakes for other key life stages is not formally reported.

This article undertakes a secondary analysis of the UK NDNS-RP delving deeper into this dataset and evaluating patterns of oily fish consumption across the life span, including early adulthood and across mid-life. A separate literature search was undertaken to derive habitual omega-3 intakes across these population groups. This novel approach will add to the evidence-base providing new insights on oily fish and *n*-3 FA intakes across the life course.

## Methods

### Survey Design

The NDNS-RP is a cross-sectional survey conducted annually since 2008 in the United Kingdom and designed to assess the diet, nutrient intake and status of the general UK population who are aged 1.5 years and older (22). The survey is conducted across all four countries of the UK and is designed to provide a representative UK population (22). Each year since 2008 the survey aimed to recruited 1,000 people per year comprised of 500 adults aged ≥19 years and 500 children aged 1.5 to 18 years (22).

The NDNS-RP adopts a stratified two-stage clustered design using post-code addresses as the Primary Sampling Units (PSUs) (22). This helps to ensure that the included sample is representative of UK population. Households are then randomly sampled within PSUs with one adult (19≥ years) and one child (1.5–18 years) being invited to participate in the survey (22). Children were asked to take part within some households to ensure that an adequate number of children is included in order to perform various comparisons (22). The present analysis uses data collected from the stage 1 interviewer visit—from the face-to-face computer assisted personal interview and 4-day food diary (22).

### Secondary Analysis

In the UK, public health guidance recommends that a healthy and balanced diet should contain two portions of fish each week of which one should be oily (i.e., herring, mackerel, pilchards, sardines, sprats, trout) (23). A portion is defined as around 140 grams when cooked (23). This guidance reflects the previously reported Scientific Advisory Committee on Nutrition (SACN) recommendations for fish and oily fish (20). Given this guidance in the present publication this was calculated to be equivalent to a daily average minimum of 40 grams of total fish, of which 20 grams should be from oily fish.

In the present analysis and NDNS-RP oily fish consumers included: “*Any oily fish or roe such as herrings, kippers, mackerel, sprats, eels, salmon, tuna (not canned), sardines, trout (baked, fried, grilled) and any homemade recipes using oily fish”* along with “*any type of oily fish purchased/retail product including canned in oil/brine/tomato, pickled, sushi, ready meals, taramasalata, pate and paste”* (24). Using this data mean average daily intakes of oily fish were calculated (oily fish + composite dishes). Including composite dishes provides a more realistic estimate as dishes with multiple food components are disaggregated into their separate ingredients (25).

The percentage of overall oily fish consumers in the UK was calculated along with the percentage with mean average daily intakes above the recommendation (≥20 g of oily fish/day). Mean daily intakes were calculated among the total UK population, and among those reporting any intake of oily fish. Similar approaches have been adopted and used in previously reported literature (26).

To assess patterns of oily fish consumption data from the UK Data Service (27) was coded and collated for key life stages including: childhood: 4–11 years, the teenage years: 12–19 years, adulthood: 20–29 years (early adulthood) and 30–39 years, mid-life: 40–49 years, 50–59 years, advancing age: 60–69 years, and ≥70 years. The analysis was also stratified by sex. In this section the analysis was exclusive to oily fish consumers. Thus, when collating the data zeros were excluded (non-oily fish consumers).

### Weighing

Survey weights provided by NDNS-RP were used in all descriptive and trend analyses to account for participant non-selection and non-response. The NDNS datasets for years 1–4, 5–6, and 7–8 were combined for analysis of years 1–8. Data was filtered to include only participants who were aged 4 years and older. Weights were recalculated after merging the datasets because there were more participants per year in years 1–4 than in years 5–6 and years 7–8. Calculations were performed as follows:

- The weight variable in each data set was divided by the sum of the weights in the respective dataset.
- Weights were multiplied by the total (combined) sum of the three weights.
- Weights were multiplied by ½ for Years 1–4, by ¼ for years 5 and 6, and by ¼ for year 7 and 8.

### Statistical Analysis

Statistical analysis was performed using Rv3.5.2. Data was weighed so that the results are representative of the UK population. The use of survey weights means that only percentages, 95% confidence intervals (CIs), are presented rather than raw frequencies. Weighed frequencies were also reported. Oily fish intake was described using the mean and 95% CIs. Categorical variables were summarized using counts and percentages Two-sample *t*-test was used to compare the average consumption of oily fish intake across males and females.

Degrees of freedom were corrected to take into account the complex survey design. Hypothesis testing was performed at percentage margin of error. Design corrected linear regression was used to assess whether there was a statistically significant linear trend in the mean consumption of oily fish intake across the following four time periods: years 1–2, 3–4, 5–6, and 7–8.

Only oily fish consumers were included in the linear trend analysis and data was adjusted for gender. The analysis was performed for all oily fish consumers and separately for each age group. Data was taken to be statistically significant if the *p*-value was ≤ 0.05.

## Habitual *n*-3 FA Intakes

A separate National Center for Biotechnology Information (NCBI) search (PubMed) was conducted to extract relevant English-language surveys published between January 2014 and March 2019.

Medical Subject Headings (MeSH) were used to filter publications. The search terms “Fatty acids”, “Omega 3” and “surveys” were used to filter publications. For inclusion studies needed to report data on habitual *n*-3 intakes. The fatty acids of interest were EPA and DHA so intakes needed to report on these. Articles were excluded if they were published before 2004, focused on omega-6 or 9 fatty acids, circulating/tissue *n*-3 status or aspects of health rather than dietary intakes.

Data extracted from each article included: (1) Author and country of research, (2) subjects (number of participants), (3) life-stage (age), (4) design and methods, (5) habitual intakes, and (6) main findings.

## Results

### Oily Fish Intakes

The full NDNS-RP data included dietary data for 12,097 UK individuals. Data was filtered to include participants who were ≥4 years leaving a total of 11,715 participants. Of these, total fish consumers represented 62.2% (weighed *n* = 7,284, = 95% CI 60.8, 63.5%) of the UK population who were 4 years or older. Oily fish consumers represented 25.2% (*n* = 2,949, 95% CI 23.9%, 26.5%) of the UK population ≥4 years.

As shown in Table 2 only a quarter (25.2%) of the entire population were “oily fish consumers.” The percentage of oily fish consumers was higher with age with the exception of those aged 40–49 years (30.3% were oily fish consumers). In childhood (4–11 years) only 12.7% consumed oily fish during the survey period. Higher intakes were observed with advancing aged with 38% of those aged ≥60 years eating some oily fish. However, oily fish consumption does not necessarily mean that recommendations are being met. Amongst those consuming fish only a quarter-−25.5% consumed ≥20 g/day of oily fish and aligned with dietary recommendations. Only 7.3, 12.8, and 15.6% of children, teenagers and those in early adulthood (20–29 years) met oily fish recommendations, respectively. Achievement of oily fish benchmarks peaked at age 60–69 years with 40.6% meeting dietary targets. When data collated oily fish consumers and non-consumers the overall percentage meeting recommendations declined to 15.9%.

**Table 2:** Oily fish consumption in the UK (percentage of consumers/those meeting recommendations).

	**Oily fish consumers, *n***	**% oily fish consumers (95% CI) (UK total)**	**% meeting oily fish recommendations (Fish consumers only)**	**% meeting oily fish recommendations (UK total—Fish consumers+ Non-consumers)**
4–11 years	143	12.7% (11.1%, 14.3%)	7.3% (5.7%, 9%)	4.4% (3.4%, 5.4%)
12–19 years	156	13.2% (10.9%, 15.5%)	12.8% (9.8%, 15.7%)	6.3% (4.9%, 7.8%)
20–29 years	274	16.4% (12.9%, 19.8%)	15.6% (11%, 20.3%)	8.8% (6.1%, 11.5%)
30–39 years	428	26.5% (23.1%, 30%)	26.9% (22.4%, 31.3%)	16.2% (13.3%, 19%)
40–49 years	410	23% (20%, 26%)	22.4% (18.5%, 26.2%)	14% (11.5%, 16.5%)
50–59 years	465	30.3% (26.7%, 33.9%)	31% (26.6%, 35.4%)	20.4% (17.3%, 23.5%)
60–69 years	509	38.2% (34.2%, 42.1%)	40.6% (35.6%, 45.5%)	28.6% (24.8%, 32.3%)
70 years +	564	38.5% (34.5%, 42.5%)	36.3% (31.9%, 40.6%)	26.1% (22.8%, 29.4%)
Total	2,949	25.2% (23.9%, 26.5%)	25.5% (23.9%, 27.1%)	15.9% (14.8%, 16.9%)

Except for UK adults in their forties oily fish intakes increased proportionately with age up to 69 years and became lower when adults reached their seventies (Table 3). Mean oily fish intakes were just 12.1 g/day—substantially lower than the ≥20 g/d benchmark used to indicate when recommendations were being met. Males aged 30–39 years consumed significantly more oily fish compared with females of this age (15.4 vs. 11 g/day *P* ≤ 0.05). Males aged 60–69 years also ate more oily fish than similarly aged women (21.8 vs. 16.6 g/day *P* = 0.049). Interestingly, in mid-life (age 50–59 years) women consumed more oily fish than men of this age (17.4 vs. 12.7 g/day *P* = 0.048).

**Table 3:** Average daily consumption of oily fish across the UK population.

	**Oily fish intake (g/day)**	**Female**	**Male**	***P***
4–11 years	3.48 (2.93, 4.04)	3.05 (2.31, 3.8)	3.88 (3.05, 4.71)	0.15
12–19 years	5.67 (4.55, 6.8)	6.06 (4.42, 7.7)	5.27 (3.77, 6.76)	0.48
20–29 years	8.64 (4.98, 12.3)	8.69 (5.85, 11.5)	8.59 (2.51, 14.7)	0.97
30–39 years	13.2 (10.8, 15.6)	11 (8.53, 13.4)	15.4 (11.5, 19.3)	0.05^*^
40–49 years	10.1 (8.42, 11.8)	9.65 (7.52, 11.8)	10.5 (7.96, 13.1)	0.6
50–59 years	15.1 (12.8, 17.4)	17.4 (14.1, 20.6)	12.7 (9.5, 15.9)	0.048^*^
60–69 years	19.1 (16.5, 21.6)	16.6 (14, 19.2)	21.8 (17.3, 26.2)	0.049^*^
70 years +	16.3 (14.3, 18.3)	21.8 (17.3, 26.2)	17.2 (13.1, 18.3)	0.48
Total	12.1 (11.3, 13)	11.8 (10.8, 12.7)	12.5 (11.1, 13.8)	0.38

*Results are summarized as mean (95% CI)*.

* *P ≤ 0.05 indicates significant differences between males and females*.

Results showed that there were no statistically significant linear trends in the mean consumption of oily fish (g/day) across the four NDNS-RP periods (*P* > 0.05) (Table 4). No statistically significant linear trend was observed within any of the age groups except for the 50–59 years age group which showed a statistically significant decreasing linear trend across the four periods (*P* < 0.05).

**Table 4:** Mean oily fish consumption (g/day) by year.

	**Years 1–2**	**Years 3–4**	**Years 5–6**	**Years 7–8**	***P* Linear trend**
4–11 years	3.37	3.64	3.4	3.53	0.454
12–19 years	4.69	3.92	8.01	5.86	0.534
20–29 years	6.83	8.89	5.96	13.01	0.137
30–39 years	12.62	10.51	15.3	14.57	0.55
40–49 years	10.49	10.72	9.38	9.84	0.08
50–59 years	18.15	13.35	15.78	12.61	0.0478^*^
60–69 years	22.03	14.71	21.15	18.52	0.135
70 years +	12.77	19.28	18.02	15.94	0.22
Total	12.3	11.3	12.8	12.1	0.43

* *P < 0.05*.

*Linear trend analysis was adjusted for sex*.

## Habitual *n*-3 FA Intakes

**Figure 2:**
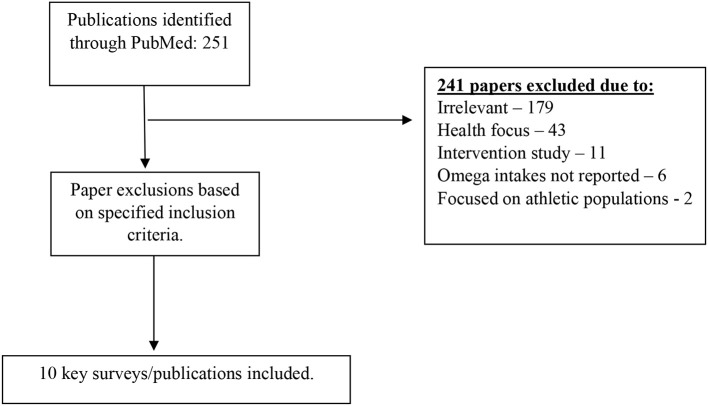
Flow diagram for database search results.

The NCBI search identified 251 publications. After the exclusion of irrelevant papers (*n* = 179), those focusing on health (*n* = 43), intervention studies (*n* = 11), those where omega-3 intakes were not reported (*n* = 6) or studies focusing on athletic populations (*n* = 2) this left ten key surveys for general review. The procedure of qualifying papers is shown in Figure 2. Of these, seven studies were conducted in the USA or Canada, one in New Zealand and two in Europe (Table 5).

**Table 5:** Surveys and key studies reporting data on habitual omega-3 intakes.

**Author (year) country**	**Subjects**	**Life stage**	**Design and methods**	**Habitual intakes (mg/d)**	**Main findings**
Thompson et al. (28) USA	*n* = 45,347	Mean−37.2 years	NHANES evaluation	EPA 32.6 DHA 64.4 EPA+DHA 96.9 (foods + supplements)	Toddlers, children and adolescents (aged 1–19) had significantly lower *n*-3 FA intake (*p* < 0.001) compared to adults and seniors, which remained significant after adjusting for caloric intake
Zhang et al. (29) USA	*n* = 11,465 CB age. *n* = 1,180 pregnant women	CB age and pregnancy	NHANES evaluation.	EPA 26.8 DHA 62.2 EPA+DHA 88.1 (foods + supplements)	Over 95% of the sample did not meet the daily intakes of 250 mg EPA and DHA. The majority of U.S. CB age and pregnant women consumed significantly lower amounts of seafood than guidelines recommend.
Bishop and Leblanc (30) Canada	*n* = 54	Pregnancy (high SES)	Cross-sectional study	66.7 and 64.8% met the FAO/WHO recommendation of 200 mg/d DHA and 300 mg/d DHA + EPA, respectively	The majority of high SES women did not meet n-3 recommendations from food alone. Continued prenatal education on the importance of fish intake and on the addition of ω-3 prenatal supplement is essential.
Nordgren et al. (31) USA	*n* = 6,478 women CB age. *n =* 788 pregnant	CB age and pregnancy	NHANES evaluation	CB age: EPA 30.2 DHA 58.3 EPA+DHA 88.5 (foods + supplements) Pregnant: EPA 34.3 DHA 66.4 EPA+DHA 100.8 (foods + supplements)	*n*-3 FA intake is a concern in pregnant women. Women of CB age in the USA and socioeconomically disadvantaged populations are more susceptible to deficiencies
Eickstaedt et al. (32) New Zealand	*n* = 596	3rd T of pregnancy	Cross-sectional study	DHA 110 (foods + supplements)	Only 30.9% of participants consumed more than 200 mg/d DHA. Those taking n-3 supplements (19.6%) were 16.5 times more likely to meet recommendations for DHA. Fish and seafood were the main contributors to DHA (84.8%) intakes, yet only 21.7% of women consumed fish at least twice per week.
Richter et al. (33) USA	*n* = 24,621 individuals.	Across the life-course	NHANES evaluation	EPA + DHA 110 (median) EPA + DHA 170 (mean) (food sources)	*n*-3 FA intake was highest in men aged 20 years+ and lowest in children and women who are or may become pregnant and/or are lactating. Only 6.2% of the total population reported n-3 FA supplement use.
Sioen et al. (34) Europe	NR	Across the life course	53 studies from 17 different European countries	EPA and/or DHA intakes were only as recommended in 26% of the countries	Intake of *n*-3 and *n*-6 PUFAs may be suboptimal in specific population groups in Europe
Forsyth et al. (35) UK	NR	Infants and children aged 6–36 months	FAO Food Balance Sheets and composition data used to generate mean per capita intake estimates	DHA 48.9 (across 76 developing countries)	Global recommendations on DHA in early life need to reflect the specific needs of infants and families living in low income countries
Keim and Branum (36) USA	2496 US children aged 12–60 months	Infants and Toddlers	NHANES evaluation	12–24 months: EPA 5 DHA 19.78 25–36 months: EPA 5.72 DHA 20.5	Children 12–24 months of age had lower total *n*-3 FA intakes than children 49–60 months of age and the lowest n6: n3 ratio, upon adjustment for energy intake
				37–48 months: EPA 6.41 DHA 20.8 49–60 months: EPA 7.18 DHA 21.0 Overall: 12–60 months: EPA 6.03 DHA 20.47 (food sources)	
Papanikolaou et al. (37) USA	*n* = 14,338	19+ years	NHANES evaluation	Male 19+ EPA 27 DHA 75 Male 19–50 years EPA 28 DHA 77 Male 51 years+ EPA 26 DHA 71 Female 19+ EPA 18 DHA 51 Female 19–50 years EPA 18 DHA 48 Female 51 years+ EPA 19 DHA 54	Males had higher (*p* < 0.05) intake of EPA and DHA from foods and dietary supplements relative to females and older adults had higher intakes of EPA, but not DHA compared to younger adults

*CB, childbearing; EPA, eicosapentaenoic acid; DHA, docosahexaenoic acid; FA, fatty acid; NHANES, National Health and Nutrition Examination Survey; NR, not reported; SES, Socio-economic status; T, trimester*.

The US NHANES (National Health and Nutrition Examination Survey) is presently one of the largest sources of information helping to ascertain intakes of EPA and DHA in the developed world. Six of the surveys identified used data from the NHANES dataset (28, 29, 31, 33, 36, 37). An updated assessment using data from years 2003 to 2014 showed that young children and adolescents had some of the lowest *n*-3 FA intakes compared to adults and seniors, whilst women tended to eat less fish and have lower *n*-3 FA intakes than males (28).

An earlier evaluation of the NHANES dataset (2003–2008) similarly found that n-3 FA intakes were highest in males but lowest in children and females, including those of childbearing age (33). In terms of dietary sources, fish was the largest dietary contributor (71.2%) of *n*-3 FAs and only 6.2 percent reported *n*-3 FA supplement use (33). Other work analyzing the same U.S. dataset (2003–2008) showed that amongst adults mean EPA and DHA intakes (23 and 63 mg/day, respectively) from food sources were substantially lower than recommendations, indicating a need for supplementation strategies (37).

Several NHANES surveys have focused on EPA and DHA intakes during the childbearing years, pregnancy and lactation periods (28, 29, 31). *n*-3 FA intakes do not appear to differ between pregnant and non-pregnant females. Thompson et al. found that pregnant women consumed less *n*-3 rich fish and intakes were “sustained” via supplements (28). Other research using NHANES data from cycles 2001–2002 to 2013–2014 reported slight increases in *n*-3 intakes over the 14-year span although most recent EPA and DHA intakes were 26.8 and 62.2 mg/day, and 95% of the population failed to meet *n*-3 benchmarks (250 mg/day) (29). Similarly, Nordgren et al. using NHANES 2003–2012 cycles found mean EPA + DHA intakes to be just 80 mg, with no evidence of change during pregnancy (31). This suggests a clear need for *n*-FA acid intakes to be increased given consistent levels of under consumption.

Other work has looked at *n*-3 FA intakes during pregnancy in New Zealand and Canada. In the New Zealand study women consuming omega-3 supplements (19.6%) were 16.5 times more likely to align with dietary recommendations for DHA (32). In Canada, a cross-sectional survey of women of high socioeconomic status showed that only 66.7% met the DHA 200 mg/d recommendation indicating that ongoing education about the importance of oily fish and *n*-3 FA supplementation is needed (30).

One survey using NHANES data (2003–2008) collated *n*-3 intakes during the early years of life. Amongst children aged 12–60 months mean EPA and DHA intakes were 6.03 and 20.47 mg/day with younger children (12–24 months) have lower average intakes than older children (49–60 months) (36). Low prevalence of fish intakes were one explanation provided for such poor omega-3 intakes (36). Forsyth et al. also conducted an evaluation of DHA intakes in infants and young children (6–36 months) and estimated that mean daily dietary intake of DHA would be around 48.9 mg/day in developing countries (35).

In the UK the NDNS-RP does not presently collate data on *n*-3 FA intakes. However, a publication written by British authors' collated data from 53 studies on EPA and DHA intakes across 17 different European countries (34). It was concluded that EPA and/or DHA intakes were only as recommended in 26% of the countries studies implying that intakes are inadequate across many parts of Europe (34).

## Discussion

Guidelines for oily fish intakes have been developed to promote adequate intakes of EPA and DHA in children and adults whilst taking on board toxicological considerations (20). There is a growing body of science that oily fish, fish-oil products and their constituent *n*-3 FAs can have extended health benefits throughout life including with respect to: fetal development, neuronal, retinal, and immune function (9).

Unfortunately, the present paper shows that oily fish is under consumed across the lifespan—a trend that is even more apparent amongst children, teenagers and young adults. Females also have a tendency toward lower oily fish intakes, as evidenced by the present analysis and others (33, 37). The explanations behind this are likely to be multi-factorial. Reported barriers to oily fish consumption have included its expense and poor satisfaction with its quality and sensory properties (38–40).

From the surveys identified EPA and DHA intakes were continually below dietary guidelines with young people, females, and pregnant mothers appearing to have some of the lowest intakes (29–32, 34, 36, 37). Shortfalls become more apparent in pregnancy when guidelines have incremental changes widening the gap between intakes and recommendations (28, 29). Unfortunately, in the UK *n*-3 FA data is not reported as part of the NDNS-RP and the addition would be of value. Should this be undertaken it would be advised that nutritional “compositional data” should be updated first. A recent publication reports that the EPA and DHA content of farmed salmon has halved due to altered fish production methods and the replacement of marine-based fish feeds with sustainable alternatives (41). Subsequently, it is tenable that EPA and DHA intakes are even lower than values reported in this review.

Confusions and misconceptions could be further hampering intakes. From surveys identified it was consistently seen that oily fish/*n*-3 FA intakes were no different between pregnant and non-pregnant women. As observed by Thompson et al. oily fish intakes even declined in pregnancy (28). This uncertainty may be due to concerns about potential contaminants such as methylmercury or lack of confidence about buying and preparing seafood (42). Ongoing trepidations about sustainability and ethics of fish consumption (43) could also potentially drive future oily fish intakes down further. Healthcare professionals can play a key role in helping to clear up confusions, particularly amongst women who are pregnant and caregivers of young children (42).

In terms of perceptions, a study of 340 family physicians showed that 51 percent believed their blood *n*-3 FA status to be “desirable” but only 5 percent had an omega-3 index ≥8 percent indicating discrepancies between perceived and actual n-3 FA status (44). Similarly, a cross-sectional study of German and U.S adults showed that more than half were aware that n-3 FAs were beneficial for heart and brain health and could provide examples of foods sources but this “knowledge” did not correlate with *n*-3 FA status (45). Amongst young adults (18–25 years) research shows that EPA and DHA abbreviations were recognized by 51 and 66%, respectively, but it was not assessed whether this knowledge led to better omega-3 profiles (46).

*n*-3 FAs appear to be particularly important at key phases of the life-cycle. In pregnancy, supplementation with brain specific fatty acids (two Equazen Mumomega capsules daily with each capsule providing 300 mg DHA, 42 mg EPA, 150 mg *n*-6 evening primrose oil and 15 mg GLA vs. oleic acid placebo) has been found to augment new-born infants' brain size (47). In particular, males born to mothers taking omega-3 capsules had larger brain volumes, total gray matter, corpus callosum and cortical volumes indicating differential sex sensitivity of fetal brain to pregnancy fatty acid profiles (47).

Observational evidence has shown that low EPA/DHA levels may be connected with rising prevalence rates of development disorders in childhood such as ADHD and dyslexia (48). Amongst those with ADHD one study found that children receiving Omega-3/6 fatty acids—Equazen—delivering 558 mg EPA, 174 mg DHA, and 60 mg Gamma Linolenic Acid (GLA) in a respective ratio of 9:3:1 over a 12-month duration did not require such a high dose of methylphenidate to manage and reduce their ADHD symptoms (0.8 mg/kg/day vs. 1.0 mg/kg/day) (49). Other research has also shown that similar *n*-3 FA Equazen supplement improved reading ability, namely “phonologic decoding time” and “visual analysis” in mainstream children and particularly those with attention problems (50). Given this, increased awareness of the importance of *n*-3 FAs is needed amongst healthcare and educational professionals.

Overall, the results of this publication demonstrate that policies are needed to improve oily fish consumption and integrate supplementation strategies alongside this. The latter becomes particularly important during life stages such as pregnancy, childhood and adolescence. Should a UK analysis of *n*-3 intakes be conducted it seems likely that the “real picture” could be more foreboding if updated food compositional data was to be used.

## Limitation and Future Directions

There are a number of limitations that may be associated with the methods adopted in the present publication. Firstly, the UK NDNS-RP only collects dietary data over a 4-day period using dietary records. It is therefore possible that some participants who were fish consumers may have simply reported during a period of 4 days when they consumed no oily fish.

Equally, when collating data on EPA and DHA there was heterogeneity between study methods e.g., dietary recalls vs. food frequency questionnaires that makes comparisons more cumbersome (34). Of these methods it has been reported elsewhere that food frequency questionnaires appear to derive suitable estimates of *n*-3 FA intakes (51). These methods now need to be applied to extended populations and used alongside or in replacement of 4-day food diaries to enable a more accurate estimate of long-term of *n*-3 intakes. Nutritional biomarkers should also ideally be used to better reflect an individual's “normal” intakes.

When extrapolating data from *n*-3 FA intake surveys a range of metrics were applied to represent the intake data. Some used the mean (arithmetic or geometric), others the median and different age cut-offs applied when calculating these. EPA and DHA data was also not always reported per 100 grams, with some reporting this as per 1,000 kcal (28). This makes direct comparisons difficult. Bearing this in mind there is a need to harmonize methodologies and the way in which data is presented.

## Conclusions

The present publication has identified that UK oily fish intakes are suboptimal across the lifespan. Some of the lowest intakes were amongst children, teenagers and adults which poses a cause for concern. This appears to be coupled with inadequate global EPA and DHA intakes. Unfortunately, declining EPA and DHA profiles of farmed fish and the movement toward plant-based diets is only likely to exacerbate this already concerning situation. Urgent public health strategies are needed to promote lipid health which should also include suitable supplementation strategies.

## Data Availability Statement

Publicly available datasets were analyzed in this study. This data can be found here: https://www.ukdataservice.ac.uk/get-data.

## Author Contributions

The author confirms being the sole contributor of this work and has approved it for publication.

### Conflict of Interest

ED is the Director of Nutritional Insight, a writing consultancy. The author declares that the research was conducted in the absence of any commercial or financial relationships that could be construed as a potential conflict of interest.
